# Association between social phobia and the risk of arrhythmia using the Korean National Sample Cohort: a retrospective cohort study

**DOI:** 10.1186/s12888-022-03689-6

**Published:** 2022-01-14

**Authors:** Hyunkyu Kim, Wonjeong Jeong, Seung Hoon Kim, Jun Ho Seo, Jin Sun Ryu, You-seok Kim, Jeong-Ho Seok, Sung-In Jang, Eun-Cheol Park

**Affiliations:** 1 Department of Preventive Medicine & Institute of Health Services Research, Yonsei University College of Medicine, 50 Yonsei-ro, Seodaemun-gu, Seoul, 03722 Republic of Korea; 2 Institute of Health Services Research, Yonsei University, Seoul, Republic of Korea; 3 Institute of Behavioral Science in Medicine, Yonsei University, Seoul, Republic of Korea; 4 Department of Psychiatry, Yonsei University College of Medicine, Seoul, Republic of Korea; 5 Department of Public Health, Graduate School, Yonsei University, Seoul, Republic of Korea; 6 Department of Hospital Administration, Graduate School of Public Health, Yonsei University, Seoul, Republic of Korea

**Keywords:** Social phobia, Arrhythmia, Retrospective cohort study, Propensity score matching

## Abstract

**Background:**

Social phobia shares symptoms with arrhythmias, such as palpitations and chest discomfort. However, it is unclear how social phobia is associated with the actual risk of arrhythmia. This study aimed to investigate whether social phobia is associated with the risk of arrhythmia using a nationally representative sample cohort.

**Methods:**

This retrospective cohort study assessed data from the 2002–2013 Korean National Health Insurance Service National Sample Cohort. Using 1:3 propensity score matching for sex, age, income, and insurance status, 1514 patients with social phobia and 4542 control group patients were included in the study. Social phobia and arrhythmia were defined per the International Classification of Diseases, 10th revision. Using cox proportional hazard regression, hazard ratios (HRs) were calculated to estimate the risk of arrhythmia in patients with social phobia.

**Results:**

There were statistically significant associations between social phobia history and elevated risks of arrhythmia. Patients with social phobia had a higher risk of arrhythmia after adjusting with covariates (HR = 1.78, 95%CI = 1.25–2.55). Among different types of arrhythmias, atrial fibrillation and flutter presented the highest risk (HR = 2.20, CI = 1.06–4.57) compared to paroxysmal tachycardia (HR = 1.07, CI = 0.39–2.91) and other cardiac arrhythmias (HR = 1.83, CI = 1.16–2.89).

**Conclusion:**

This study identified the association between social phobia and the risk of arrhythmia in a South Korean representative cohort. These results suggest that social phobia should be treated properly to reduce arrhythmia risks.

## Background

Psychiatric disorders and psychological conditions have been associated with the occurrence of physical illness [1–4]. It is well-proven that physical diseases, such as strokes, Parkinson’s disease, and heart diseases, cause psychiatric symptoms such as depression, anxiety, or sleep disturbance [5–7]. Conversely, the hypothesis that mental illness causes physical illness has also been investigated. Particularly, hypotheses have posited that anxiety disorders can cause physical symptoms, such as palpitations, chest pain, and difficulty in breathing, and studies continue to examine these relationships [8, 9]. Previous research has shown that generalized anxiety and panic disorders are associated with risks of coronary heart disease and other cardiovascular diseases [10–12]. These studies suggest that further associations between other types of anxiety disorders and physical illnesses might exist.

Among various anxiety disorders, social anxiety disorder or social phobia is one of the most common and distressing conditions, wherein a person feels severe anxiety and associated physical symptoms in social situations [13, 14]. Cultural contexts affect the prevalence of this disease; yet, its lifetime prevalence is considered to be higher than 10% [15–17]. However, since these symptoms occur in specific social situations, many patients simply avoid the same and rarely utilize mental health management or treatment services [18]. Nevertheless, simply avoiding situations to eliminate experiencing psychological and physiological symptoms does not cure social phobias, for which continuous cognitive behavior therapy, psychotherapy, and medication are required. Among physiological symptoms, many patients with social anxiety disorder have reported experiencing palpitations that feel like arrhythmia. However, studies have not yet determined whether social anxiety disorder causes arrhythmia, necessitating investigation and validation of the same through large-scale cohort studies. Relevant results could serve as the basis for improving treatments for both arrhythmia and social anxiety disorder.

This study aimed to investigate the association between social phobia and risk of arrhythmias, as well as the differences among various types of arrhythmia among patients with social phobia, using Korean nationwide claims data.

## Methods

### Study population and data

The data analyzed in this study were acquired from the Korean National Health Insurance Service National Sample Cohort (NHIS-NSC) of 2002 and 2013, from the National Health Insurance Service (NHIS). The Korean NHIS provides researchers with all data of claims collected under the NHIS for academic investigation and policymaking. Since universal healthcare coverage was achieved in 1989, the Korean NHIS has been collecting all claims data from all cases of South Korean medical utilization. Among the 47,851,928 individuals in 2002, 46,605,433 individuals were selected after excluding non-citizens, for constructing the sample cohort. From the full NHIS database, the representative sample cohort was constructed with 1,025,340 individuals, comprising 2.2% of the total South Korean population by random stratification [19]. Participants from the cohort were followed up with and all visiting data were recorded in a database; those who passed away or migrated were excluded. Moreover, those who had sought treatment for social phobias or arrhythmia within a year of the washout period were also excluded, as were individuals diagnosed with arrhythmia within a year of social phobia diagnoses. After exclusion, 1514 individuals were selected as the social phobia group.

The NHIS-NSC database includes information on socioeconomic status and codes from the clinically determined International Classification of Diseases, 10th revision (ICD-10). Since we used the de-identified these data, the need for informed consent was waived by the Institutional Review Board of Yonsei University’s Health System (4–2021-0155).

Participants of the social phobia group were then matched with control group participants (who had not been previously diagnosed with social phobia or arrhythmia) via propensity score matching. The propensity score was derived using logistic regression to calculate the probability of social phobia with covariates of sex, age, insurance, and income status. After calculating the propensity score, 1:3 greedy matching was performed using the OneToManyMTCH macro for SAS [20].

### Study variables and covariates

The classification of social phobia was based on the diagnostic ICD-10 codes F40.1. Participants’ first dates of diagnosis with F40.1 by doctors were regarded as the diagnoses dates for social phobia. Participants with ICD-10 codes I47, I48, and I49 were considered to have arrhythmias. For each participant, we identified the date of diagnosis of arrhythmia or the end of the study period (December 30, 2013) as the final follow-up date. Various demographic variables that might affect the occurrence of social phobia or arrhythmia, such as age, sex, social security status, residential region, disability, income status, and the Charlson comorbidity index (CCI), were included in the regression model as covariates. CCI score is the index for assessing the participants’ comorbidities which can alter the risk of mortality for use in longitudinal studies. The score was calculated by weighting 1 ~ 6 scores for 19 comorbid diseases. The comorbid disease categories included in CCI score consists of myocardial, vascular, pulmonary, endocrine renal, gastrointestinal, cancer/immune and neurologic comorbid diseases [21]. Dementia is the only psychiatric comorbid disease included in the CCI evaluation and categorized into neurologic comorbid disease. We used the ICD-10 codes for each comorbid disease to calculate the CCI score of participants [22].. Participants were divided into three groups according to their CCI scores: 0–2, 3–4, ≥5. The diagnoses of diabetes, hypertension, chronic obstructive pulmonary disease (COPD) and hyperthyroidism obtained by using ICD-10 codes were also included in the analysis. Age was divided into seven 10-year groups (0–9, 10–19, 20–29, 30–39, 40–49, 50–59, ≥60) to distinguish between risks of arrhythmias among age groups. Region was divided into three categories according to population density: metropolitan, city, and rural. Social security status was categorized by participants’ health insurance status as per the criteria of South Korea’s National Health Insurance system within which self-employed or privately employed individuals are covered by National Health Insurance. Medical aid beneficiaries consist of individuals who have an income below the government-defined poverty threshold, or disabilities which enable them to receive free inpatient and outpatient care through the government. For the dependent variable subgroup analysis, arrhythmias were sub-grouped into three different categories: paroxysmal tachycardia (I47), atrial fibrillation/flutter (I48), and other cardiac arrhythmias (I49).

### Statistical analysis

We first examined the frequencies and percentages of each categorical variable at each participant’s baseline, and performed chi squared tests to examine the distribution of arrhythmias according to each variable. Consequently, a Cox proportional hazards model was generated for examining the association between social phobia and the risk of arrhythmia. All Cox proportional hazard models were fully adjusted with the covariates presented in Table 1. We divided participants with arrhythmia into three groups by their ICD-10 codes, and included as the outcome variables comparing to patients without arrhythmia in Cox model to investigate the risk of each type of arrhythmia. Social phobia history as exposure variable and all other covariates and were included in the analysis. Kaplan–Meier survival curves were used to compare survival rates among the groups. The results were presented as hazard ratios (HRs) with 95% confidence intervals (95% CIs) to compare the risk of arrhythmia. All analyses were conducted using the SAS software version 9.4 (SAS Institute, Cary, North Carolina, USA), and R version 4.0.3. Statistical significance was determined by a two-tailed test with a *p* value of 0.05 as the threshold.

**Table 1:** Baseline characteristics of the study population and the results of chi-square test with the Social phobia history and risk of arrhythmia

	Total		Social Phobia				***P***-value	Risk of Arrhythmia				***P***-value
			Yes		No			Yes		No		
	6056	(100.0)	1514	(25.0)	4542	(75.0)		185	(3.1)	5871	(96.9)	
**Social phobia**												0.698
Yes	1514	(25.0)						44	(2.9)	1470	(97.1)	
No	4542	(75.0)						141	(3.1)	4401	(96.9)	
**Gender**							1.000					0.635
Male	3268	(54.0)	817	(25.0)	2451	(75.0)		103	(3.2)	3165	(96.8)	
Female	2788	(46.0)	697	(25.0)	2091	(75.0)		82	(2.9)	2706	(97.1)	
**Age**							0.7079					< 0.0001
0–9	496	(8.2)	124	(25.0)	372	(75.0)		3	(0.6)	493	(99.4)	
10–19	1712	(28.3)	452	(26.4)	1260	(73.6)		27	(1.6)	1685	(98.4)	
20–29	1556	(25.7)	365	(23.5)	1191	(76.5)		48	(3.1)	1508	(96.9)	
30–39	1284	(21.2)	321	(25.0)	963	(75.0)		40	(3.1)	1244	(96.9)	
40–49	696	(11.5)	174	(25.0)	522	(75.0)		42	(6.0)	654	(94.0)	
50–59	212	(3.5)	53	(25.0)	159	(75.0)		13	(6.1)	199	(93.9)	
≥ 60	100	(1.7)	25	(25.0)	75	(75.0)		12	(12.0)	88	(88.0)	
**Social Security**							1.000					0.8458
Health Insurance	5968	(98.5)	1492	(25.0)	4476	(75.0)		182	(3.0)	5786	(97.0)	
Medical Aid	88	(1.5)	22	(25.0)	66	(75.0)		3	(3.4)	85	(96.6)	
**Region**							0.0001					0.5237
Metropolitan	2638	(43.6)	666	(25.2)	1972	(74.8)		82	(3.1)	2556	(96.9)	
City	1713	(28.3)	479	(28.0)	1234	(72.0)		46	(2.7)	1667	(97.3)	
Rural	1705	(28.2)	369	(21.6)	1336	(78.4)		57	(3.3)	1648	(96.7)	
**Disability**							0.1097					0.6491
Yes	76	(1.3)	13	(17.1)	63	(82.9)		3	(3.9)	73	(96.1)	
No	5980	(98.7)	1501	(25.1)	4479	(74.9)		182	(3.0)	5798	(97.0)	
**Income**							0.0648					0.6238
Low	647	(10.7)	185	(28.6)	462	(71.4)		21	(3.2)	626	(96.8)	
Middle	2381	(39.3)	596	(25.0)	1785	(75.0)		78	(3.3)	2303	(96.7)	
High	3028	(50.0)	733	(24.2)	2295	(75.8)		86	(2.8)	2942	(97.2)	
**Hypertension**							< 0.0001					< 0.0001
Yes	850	(14.0)	259	(30.5)	591	(69.5)		81	(9.5)	769	(90.5)	
No	5206	(86.0)	1255	(24.1)	3951	(75.9)		104	(2.0)	5102	(98.0)	
**Diabetes**							0.6514					0.0009
Yes	393	(6.5)	102	(26.0)	291	(74.0)		23	(5.9)	370	(94.1)	
No	5663	(93.5)	1412	(24.9)	4251	(75.1)		162	(2.9)	5501	(97.1)	
**COPD**							< 0.0001					0.0622
Yes	2963	(48.9)	815	(27.5)	2148	(72.5)		103	(3.5)	2860	(96.5)	
No	3093	(51.1)	699	(22.6)	2394	(77.4)		82	(2.7)	3011	(97.3)	
**Hyperthyroidism**							< 0.0001					< 0.0001
Yes	270	(4.5)	105	(38.9)	165	(61.1)		20	(7.4)	250	(92.6)	
No	5786	(95.5)	1409	(24.4)	4377	(75.6)		165	(2.9)	5621	(97.1)	
**CCI**							< 0.0001					< 0.0001
0–2	4249	(70.2)	979	(23.0)	3270	(77.0)		91	(2.1)	4158	(97.9)	
3–4	1476	(24.4)	429	(29.1)	1047	(70.9)		68	(4.6)	1408	(95.4)	
≥ 5	331	(5.5)	106	(32.0)	225	(68.0)		26	(7.9)	305	(92.1)	

Values are presented as number (%). *COPD* Chronic obstructive pulmonary disease, *CCI* Charlson Comorbidity Index

## Results

The sample’s baseline characteristics are presented in Table 1. A total of 6056 participants were included in the analysis, with 3.1% of them diagnosed with arrhythmia. The chi-squared test showed no statistical difference in the prevalence of arrhythmia between groups divided by the diagnosis of social phobia. Further, gender, social security status, region, disability, and income were also not associated with arrhythmia. Contrastingly, the univariate analyses revealed that age, hypertension, diabetes, and CCI had statistically significant effects on the risk of arrhythmia.

**Fig. 1:**
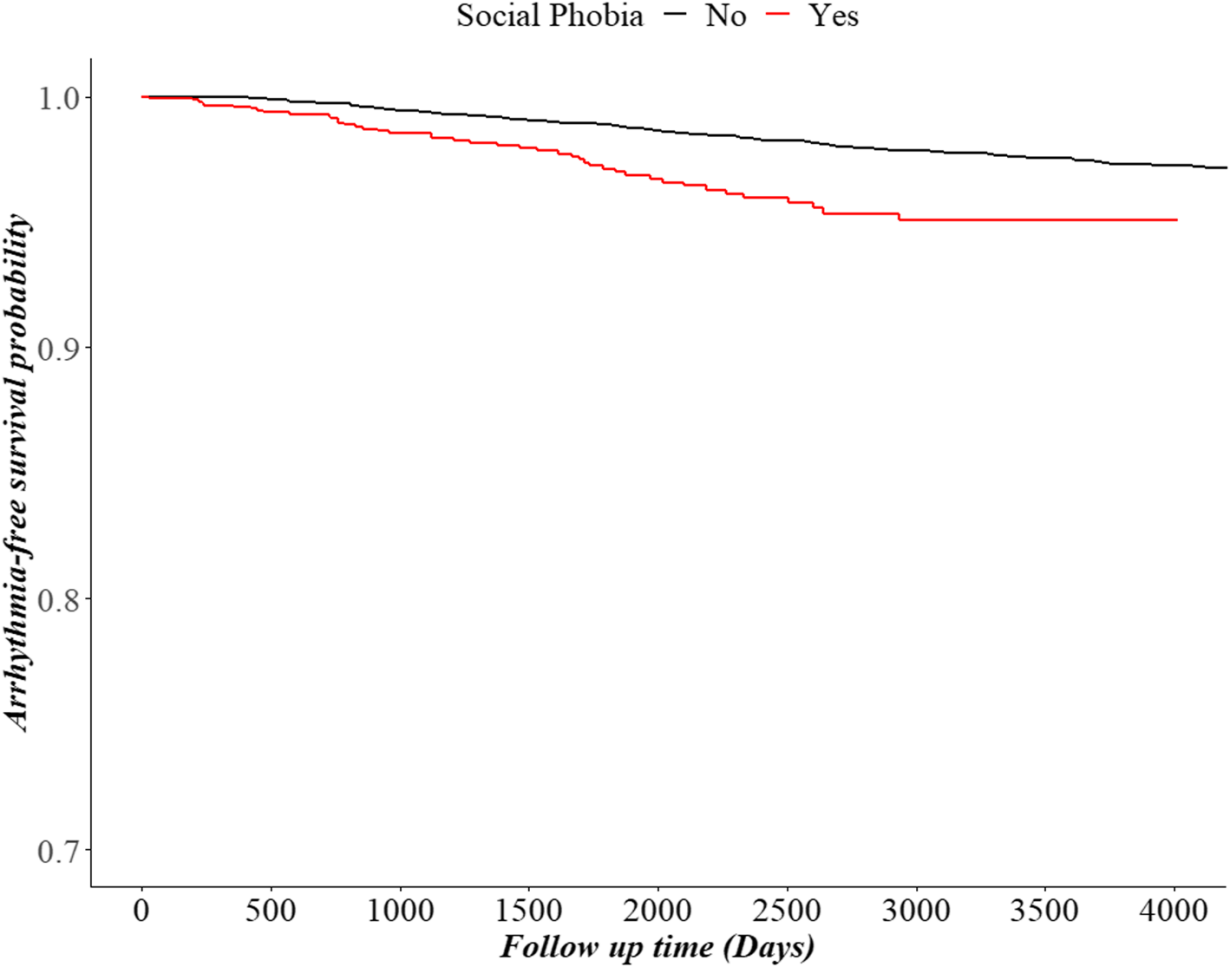
Kaplan–Meier Curve for the risk of arrhythmia divided by the positive history of social phobia

Table 2 shows the results of the survival analysis using the Cox proportional hazards regression model. Unlike results from the univariate analysis, individuals with social phobia had 1.78 times higher risks of arrhythmia after adjusting with covariates (HR = 1.78, 95% CI = 1.25–2.55). Hypertension was also a statistically significant factor affecting the risk of arrhythmia (HR = 3.50, 95% CI = 2.46–5.00), as was hyperthyroidism (HR = 1.87, 95%CI = 1.15–3.04). In contrast, gender, social security status, region, disability, diabetes, COPD, and income were not associated with the risk of arrhythmia. The results of the Kaplan–Meier curves for the sample are shown in Fig. 1. Participants with social phobia showed lower survival probability without arrhythmia as compared to those without social phobia.

**Table 2:** Results of the Cox Proportional Hazard Regression analysis on the association between social phobia and the risk of Arrhythmia

Variables	Risk of Arrhythmia				
	HR	95% CI			***P***-value
**Social Phobia**					
Yes	1.78	(1.25	–	2.55)	0.0016
No	1.00				
**Gender**					
Male	1.00				
Female	0.91	(0.67	–	1.23)	0.5382
**Age**					
0–9	0.30	(0.09	–	0.97)	0.0440
10–19	0.65	(0.40	–	1.05)	0.0797
20–29	1.00				
30–39	0.81	(0.53	–	1.24)	0.3313
40–49	1.16	(0.73	–	1.83)	0.5380
50–59	0.87	(0.45	–	1.69)	0.6785
≥ 60	1.52	(0.73	–	3.15)	0.2624
**Social Security**					
Health Insurance	1.00				
Medical Aid	1.22	(0.35	–	4.26)	0.7526
**Region**					
Metropolitan	1.00				
City	0.82	(0.57	–	1.18)	0.2913
Rural	0.99	(0.70	–	1.39)	0.9378
**Disability**					
Yes	0.73	(0.23	–	2.34)	0.5983
No	1.00				
**Income**					
Low	1.00				
Middle	1.01	(0.61	–	1.70)	0.9574
High	0.82	(0.49	–	1.38)	0.4517
**Hypertension**					
Yes	3.50	(2.46	–	5.00)	< 0.0001
No	1.00				
**Diabetes**					
Yes	0.81	(0.51	–	1.30)	0.3852
No	1.00				
**COPD**					
Yes	0.98	(0.70	–	1.37)	0.9081
No	1.00				
**Hyperthyroidism**					
Yes	1.87	(1.15	–	3.04)	0.0113
No	1.00				
**CCI**					
0–2	1.00				
3–4	1.54	(1.07	–	2.23)	0.0214
≥ 5	1.61	(0.94	–	2.74)	0.0805

All variables were included in Cox proportional hazard model. *COPD* Chronic obstructive pulmonary disease, *HR* Hazard ratio, *CI* confidence interval, *CCI* Charlson Comorbidity Index

After subgrouping arrhythmia into three groups by ICD-10 codes, atrial fibrillation and flutter (I47) showed the highest risk of arrhythmia (HR = 2.20, 95% CI==1.06–4.57) as compared to Paroxysmal tachycardia (I48, HR = 1.07, 95% CI = 0.39–2.91) and other cardiac arrhythmias (I49, HR = 1.83, 95% CI = 1.16–2.89) (Table 3).

**Table 3:** Subgroup analysis of arrhythmia by ICD-10 code and association with social phobia

	Paroxysmal tachycardia (I47)				Atrial fibrillation and flutter (I48)				Other cardiac arrhythmias (I49)			
	HR	95% CI			HR	95% CI			HR	95% CI		
**Social Phobia**	1.07	(0.39	–	2.91)	2.20	(1.06	–	4.57)	1.83	(1.16	–	2.89)

All variables in Table 1 were included in Cox proportional hazard model. The control group who had no social phobia history was used as reference in the model. *HR* Hazard ratio, *CI* Confidence interval

## Discussion

This longitudinal study found that diagnoses of social phobia are associated with elevated risks of arrhythmia among a South Korean national sample. Furthermore, among the types of arrhythmias, atrial fibrillation and flutter were most significantly associated with social phobias.

To our knowledge, this study is the first to investigate the association between social phobia and the risk of arrhythmia in a nationwide sample. Few previous studies have attempted to establish the connection between anxiety disorders and arrhythmia, and their results have generally agreed with ours. For instance, a study with a three-year follow-up period suggested that phobic anxiety was associated with ventricular arrhythmias (Odds ratio = 1.40) [23]. Several studies have revealed that anxiety after cardiac intervention or surgery is associated with an elevated risk of arrhythmia [24, 25]. However, no study has investigated the relationship between social phobia and arrhythmia. Only a few studies have examined the relationship between social phobia and other cardiac diseases [26], but their variables were limited to hypertension. The lack of research on phobia is could be due to the underestimation of the disease by patients and clinicians. Patients with social phobia tend to avoid situations that provoke their anxiety symptoms, potentially reducing their intentions to seek proper treatment such as cognitive behavior therapy or medication. Similarly, clinicians and researchers might underestimate social phobia because it is regarded as a less severe condition as compared to severe depression or bipolar disorders. However, as our results suggest, social phobia might affect serious physiological conditions, including arrhythmia. Thus, proper treatment for patients with social phobia with comprehensive education and applied research should be provided to reduce any relevant complications.

How social anxiety affects the emergence of arrhythmia could not be determined; however, several possible hypotheses exist. The results of our subgroup analysis suggest that social phobia or anxiety are associated with higher risks of arrhythmias. The dependent subgroup analysis showed that atrial fibrillation and flutter had the highest and most statistically significant hazard ratios associated with social phobia history. Atrial fibrillation is more prevalent among older age groups; thus, aging is considered as one of the risk factors for atrial fibrillation [27, 28]. Higher heart rates and overstimulation of the sympathetic nervous system might produce early aging or structural changes of the heart, which can induce atrial fibrillation or other arrhythmias. Moreover, anxiety could alter the autonomic nervous system and induce a different pattern of sympathetic activity which can increase the risk of arrhythmia [29, 30]. Another hypothesis is that patients with anxiety might recognize or detect heart symptoms earlier than others. Thus, their diagnoses of cardiac diseases could be higher than those of individuals without anxiety [31]. Patients with anxiety disorders usually have symptoms of palpitation or chest discomfort; moreover, they tend to visit clinics to test their physical conditions, which might increase the diagnosis rate of arrhythmia among such anxious. Further research should investigate the mechanisms linking social phobia and arrhythmias.

This study has several limitations. First, as we used a retrospective cohort, we could not include all variables that might affect arrhythmia development, such as family history, exercise status, height, weight, and health behaviors including smoking and drinking. Also, all detailed drug history and other disease history that might affect the incidence of arrhythmia were not included in the analysis. Additionally, the confounding variables included in our analyses could have changed throughout the 12-year study period. Thus, we applied the propensity score matching for the study population to reduce the effects of confounding variables. Second, the number of patients diagnosed with social phobia might be underestimated in the analysis. The prevalence of social phobia varied between 1.9 and 20.4% across different levels of distress [32]. However, less than 0.17% of the sample had visited clinics during the study period. Patients suffering from social phobia symptoms may be reluctant to receive treatment for psychiatric disorders, as they are often regarded as cultural taboos in Korea. Therefore, the number of patients with social phobia analyzed could be lower than the actual population with social phobia. Third, the accuracy of diagnostic information may be limited due to the inaccuracy of claims diagnoses, as suggested by a previous study [33]. To enhance diagnosis accuracy, we investigated the primary and secondary diagnostic codes and included both in the analysis. Fourth, we could not evaluate the symptom severity of social anxiety and arrhythmias. As we only defined participants using the ICD-10 codes, we could not assume the severity of the symptoms, and thus, the dose-relationship could not be established. Finally, due to the retrospective nature of the data, causal relationships could not be established. The increased risk of arrhythmia might be a temporal occurrence rather than a causal relationship.

Despite these limitations, our study possesses certain strengths. Although using claims data has its limitations, we used national cohort data, which represented the general population of Korea. The results of our study can be generalized to the entire population of South Korean individuals or other countries with similar demographic characteristics, and provide a background for the management of social phobia to curb risks of arrhythmia. This study also applied propensity score matching to reduce confounding between patients with social phobia and the control group. Moreover, since we only included patients with new-onset social phobia, this study provides additional evidence that new-onset social phobia is a risk factor for arrhythmia.

## Conclusion

In conclusion, this was the first study to identify the relationship between the history of social phobia and the risk of arrhythmia among a South Korean national representative cohort. Individuals diagnosed with social phobia are at 1.84 times higher risk of arrhythmia. Furthermore, atrial fibrillation and flutter are most strongly associated with social phobia. While risks presented by social phobia are often underestimated as it is easy to avoid relevant triggering situations, proper treatment may prevent the elevation of arrhythmia risks. Further research using prospective designs to assess the causality of social phobia-induced arrhythmia in a controlled environment should be conducted to validate these findings.

## Data Availability

The Korean National Health Insurance Service–National Sample Cohort is a public, open-access database. It is based on the health insurance claim data of all Koreans, and the sample cohort is available for public purposes and scientific research. The authors do not have permission to share these data. The sample cohort data are available after acceptance of approval for use by the national health insurance service. (https://nhiss.nhis.or.kr/bd/ab/bdaba000eng.do).
